# White-tailed eagle (*Haliaeetus albicilla*) as the definitive host of *Sarcocystis lutrae* in the Czech Republic

**DOI:** 10.3389/fvets.2022.981829

**Published:** 2022-08-18

**Authors:** Ondřej Máca, David González-Solís

**Affiliations:** ^1^Department of Pathology and Parasitology, State Veterinary Institute Prague, Prague, Czechia; ^2^Department of Zoology and Fisheries, Faculty of Agrobiology, Food and Natural Resources, Czech University of Life Sciences Prague, Prague, Czechia; ^3^Department of Systematics and Aquatic Ecology, El Colegio de la Frontera Sur, Chetumal, Quintana Roo, Mexico

**Keywords:** birds of prey, carnivores, wildlife, genetic characterization, protozoan, oocysts and sporocysts, small intestine, Czech Republic

## Abstract

The white-tailed eagle, *Haliaeetus albicilla*, has been involved in the life cycle of several *Sarcocystis* species as the intermediate and definitive host. To date, it has been supposed that the eagle might play the role as the definitive host for *S*. *Lutrae*, and, herein, we tried to elucidate it based on morphometric and molecular analyses. One out of two eagles harbored oocysts (17.0−17.4 × 11.3–11.9 μm) and sporocysts (11.3–12.3 × 8.3–9.3 μm) in the intestinal mucosa, whose sequences at *18S* rRNA, *28S* rRNA, ITS1, and *cox1* showed similar identity (97.64–100%) to published sequences of *S*. *lutrae* from other hosts. The presence of sporulated oocysts in the lamina propria of villi confirms that *S*. *lutrae* truly infects the white-tailed eagle. The white-tailed eagle is confirmed as the definitive host of *S*. *lutrae* in the Czech Republic.

## Introduction

Apicomplexan parasites of the genus *Sarcocystis* have an obligatory two-host life cycle, where some herbivores, omnivores or carnivores act as intermediate hosts and carnivores as definitive hosts. Birds of prey are mainly documented as definitive hosts for many species of protozoans of this genus, although they can also act as intermediate hosts (1, 2). One of the bird species playing both roles is the white-tailed eagle (*Haliaeetus albicilla*), which has been reported as the definitive host for *S*. *arctica, S*. *halieti*, and *S*. *lari* and as the intermediate host for an unknown *Sarcocystis* sp. and *S*. *wobeseri*-like sarcocysts (2–4). This top diurnal raptor is distributed throughout the Palearctic and preys mainly on fish, birds, and mammals (Canidae, Mustelidae, and Procyonidae) (5–8), which have been involved in the life cycle of *S*. *lutrae* as intermediate hosts (9–11) and whose definitive hosts remain uncertain.

Gjerde and Josefsen (9) suggested that the white-tailed eagle is the potential definitive host of *S*. *lutrae*, because of the geographical location of preys (e.g., otter, arctic fox), which serve as intermediate hosts. Therefore, the main goal of this work was to elucidate the role of *H*. *albicilla* as the definitive host for *S*. *lutrae* in the Czech Republic.

## Methods

Two dead, wild white-tailed eagles were sent to the State Veterinary Institute Prague by a costumer for determination of the cause of death. Eagles were necropsied and, subsequently, parasitologically examined for the presence of intestinal protozoans of the genus *Sarcocystis*. One female eagle, 4.1 kg in weight, came from the Liberec region, Czech Republic (negative to *Sarcocystis*) and the other female, 4 kg in weight, from Ústí nad Labem region, Czech Republic (positive to *Sarcocystis*). A Leica DM2500 LED optical microscope, a digital camera Leica DFC420, and microscope software Leica Application Suite X (Leica Microsystems, Wetzlar, Germany) were used for light microscopical examination. Scrapings of some parts of the intestinal mucosa (covered duodenum, jejunum, and ileum) were examined by wet mounts; thereafter, eight new scrapings were randomly collected to represent the whole intestine, transferred to separate Eppendorf tubes and used for further identification by molecular analyses. For a histological study, two tissue samples of ileum were fixed in 10% formalin, embedded in paraffin, and stained with haematoxylin and eosin staining. No skeletal muscles were examined for parasites.

Total genomic DNA was extracted from eight intestinal mucosa scraping samples by glass bead disruption using the QIAamp^®^ Fast DNA Stool Mini Kit (Qiagen, Hilden, Germany), following the instructions of the manufacturer, and purified DNA was stored at −20°C until use in polymerase chain reaction (PCR). PCR was carried out by using primers for *18S* rRNA (ERIB1/A2R, A1F/S2r, A2F/Primer BSarc) (3, 12–14), *28S* rRNA (KL_P1F/KL_P2R) (14), the ITS1 region (ITS-F/ITS-R) (15), and *cox1* (SF1/SR5) (16), with recommended annealing temperatures depending on the primer pair. All the samples were initially characterized at the ITS1 region and showed to be similar, and then two of them were also characterized at the other 3 loci. Each PCR mixture contained 12.50 μl of GoTaq^®^ G2 Green Master Mix (Promega, Madison, WI, USA), 4 μM of each primer, a 5-μl DNA template, and nuclease-free water to a total volume of 25 μl. The PCR conditions consisted of initial denaturation at 95°C for 5 min, 35 cycles of 95°C for 30 s, 52–60°C for 30 s, 72°C for 1 min, and then a final extension step at 72°C for 10 min. The amplification products were resolved on 1.5% agarose gels and visualized by ethidium bromide staining. The PCR products were purified using the ExoSAP-IT™ Express PCR Product Cleanup Reagent kit (Thermo Fisher Scientific, Waltham, MA, USA) and then directly sequenced in both forward and reverse directions using the same primers as for PCR through the commercial company Eurofins Genomics (Ebersberg, Germany). These sequences were assembled and manually edited using FinchTV software (Geospiza Inc., Seattle, WA, USA), followed by BLAST and deposited in the GenBank database under accession numbers (*18S* rRNA: ON796570; *28S*rRNA: ON796572; ITS1: ON806939; *cox1*: ON805825).

## Results and discussion

**Figure 1:**
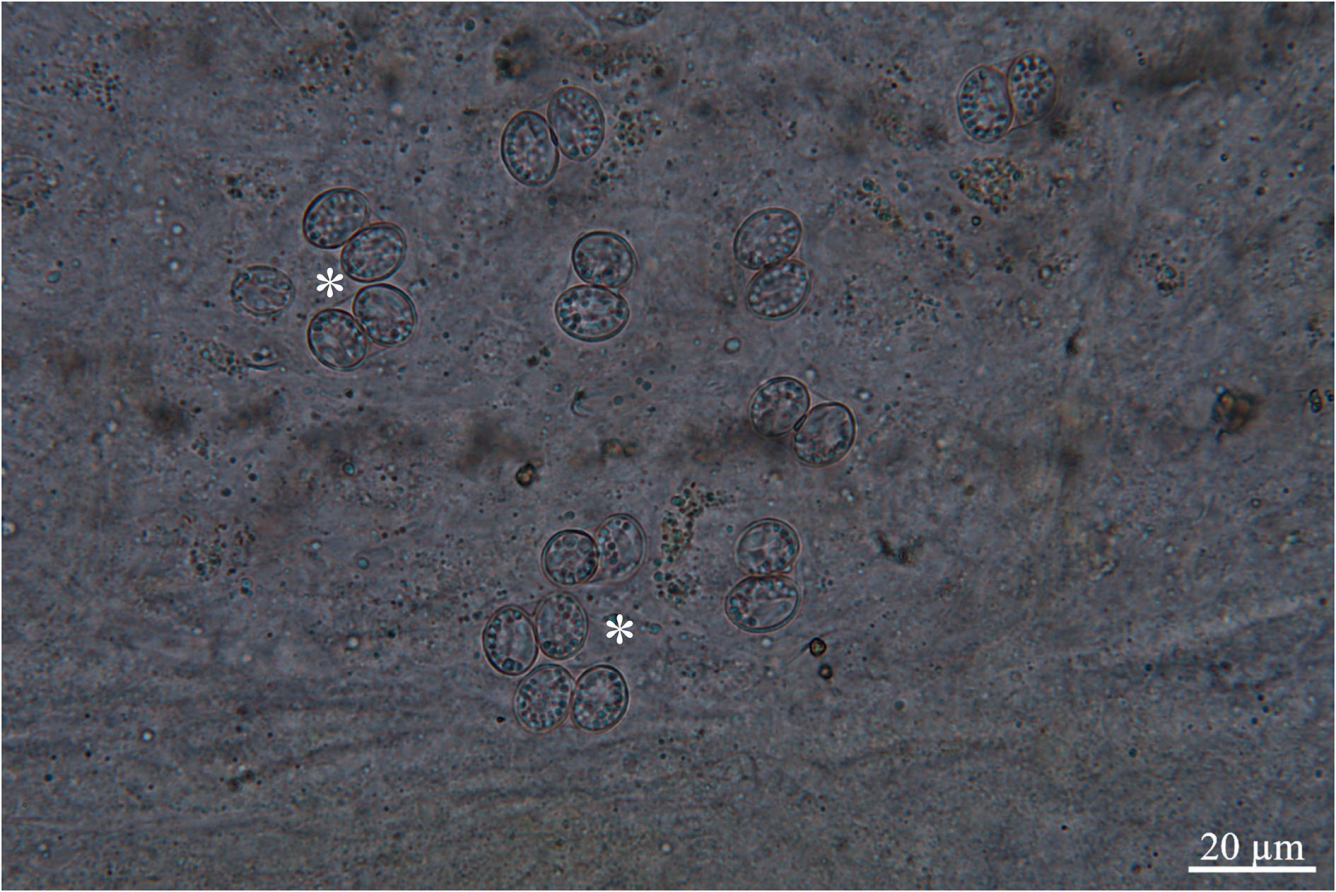
Scraping of intestinal mucosa, asterisks showing oocysts, ligth micrcosocpy.

One of two white-tailed eagles harbored oocysts/sporocysts in the mucosa throughout the small intestine (Figure 1). Oocysts (*n* = 5) were 17.0−17.4 × 11.3–11.9 μm in size, while sporocysts (n = 50) were 11.3–12.3 × 8.3–9.3 μm in size, with wall thickness of 0.5 μm. The morphological and morphometrical parameters of these developmental stages are unreliable for distinguishing species [see (3, 4, 17)]; however, after comparing the present oocyst and sporocysts with those of the other three species reported in *H*. *albicilla* (i.e., *S*. *arctica* and *S*. *halieti*/*S*. *lari*), they are slightly smaller (17.0−17.4 × 11.3–11.9 μm vs. 18.5–18.8 × 11.6–14.0 and 21.8–22.8 × 16.0−17.0 μm, respectively; sporocysts 11.3–12.3 × 8.3–9.3 μm vs. 10.6–12.7 × 8.7–10.6 and 16.0−17.0 × 10.5–11.2 μm, respectively [see (3, 4)]. The three sections of the intestine were positive to oocysts/sporocysts, with the highest intensity at ileum, followed by jejunum and duodenum.

The successful molecular analyses helped clarify that 8 samples, from the only parasitized white-tailed eagle, at ITS1 were 100% identical in the overlapping region of 338 bp (nucleotides 8–345) and belonged to *S*. *lutrae* (e.g., GenBank Nos. MG372108 and MG372109). Due to the intraspecific genetic differences at this region, double peaks occurred at some positions (449 and 532 A/G; 753 G/C) but allow to obtain up to 1,058 bp (two samples with 100% identical sequences), which is good for distinguishing species, thus cloning was unneeded. Thus, our ITS1 published sequence (1,058 bp, GenBank: ON806939) was 99.72 and 98.20% similar to *S*. *lutrae* found in the intermediate hosts, European badger (GenBank: MG372108) and otter (GenBank: MG372109), respectively, from the Czech Republic, and 97.64% to all sequences in various other intermediate hosts published in GenBank. Conversely, sequences of 338 bp were 98.76–100% to all *S*. *lutrae*-published sequences at GenBank. Even though the ITS1 region is one of the most used markers for those species of *Sarcocystis* using birds and carnivores as intermediate hosts, the *28S* rRNA gene might also be an alternative genetic marker [see (1, 17)] due to its non-intraspecific variation for *S*. *lutrae*.

An identical pattern occurred with sequences of *18S*rRNA (1,697 bp, GenBank: ON796570), *28S*rRNA (1,501 bp, GenBank: ON796572), and *cox1* (1,055 bp, GenBank: ON805825) genes, which were 100, 99.9–100, and 100%, respectively, similar to published sequences of *S*. *lutrae* at GenBank. *Sarcocystis lutrae* was originally reported in *Lutra lutra* and *Vulpes lagopus* from Norway [see (9)]. Thereafter, it has been found in *L*. *lutra, Martes foina, Meles meles, Mustela putorius, Neovison vison* (all Mustelidae), *Nyctereute sprocyonoides, V*. *lagopus, V*. *vulpes* (all Canidae), and *Procyon lotor* (Procyonidae) from the Czech Republic, Latvia, Lithuania, and Scotland (10, 18–22). All these hosts represent intermediate hosts; thus, this is the first record of *S*. *lutrae* in *H*. *albicilla* and the role of the latter as its definitive host.

**Figure 2:**
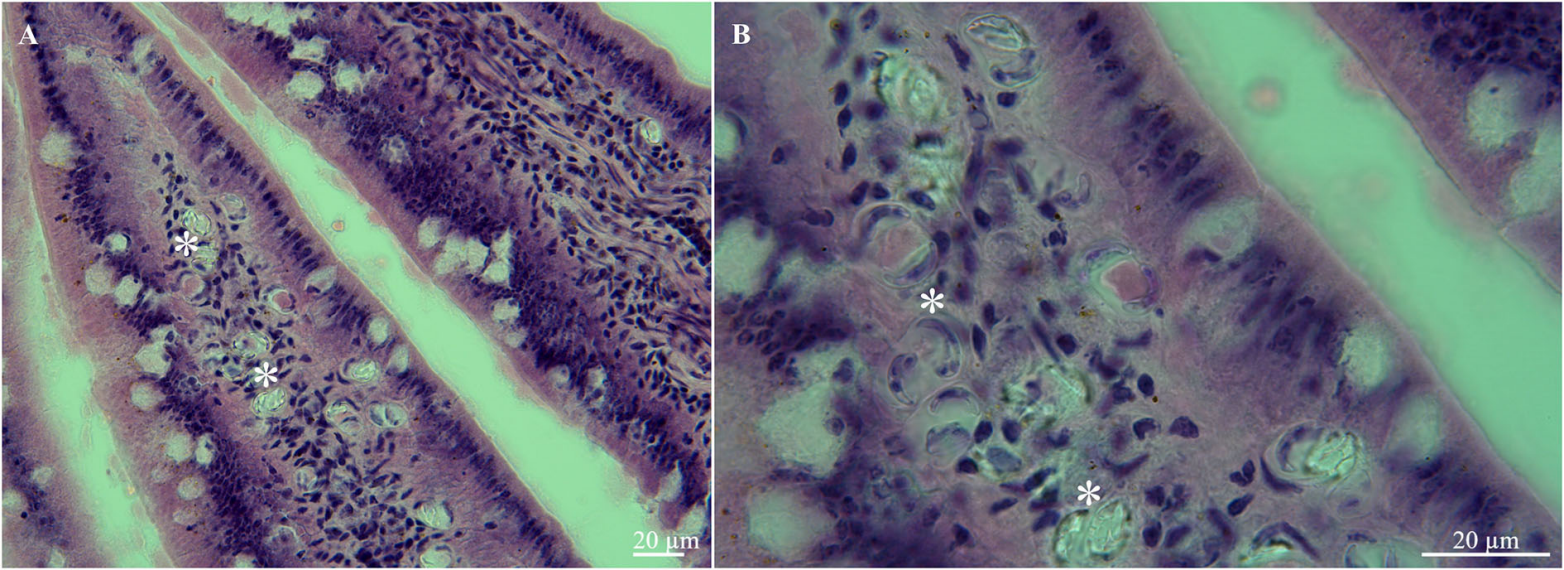
Histological tissue sections of small intestine from the white-tailed eagle (*Haliaeetus albicilla*), **(A)** lamina propria of villi with multiple oocysts (asterisks), **(B)** same, higher magnification.

The histopathological analysis of the small intestine showed the massive presence of sporulated oocysts in the lamina propria of villi (Figures 2A,B). This finding confirms that *S*. *lutrae* truly infects the white-tailed eagle and that the oocysts/sporocysts in the intestinal mucosa are not only part of the feeding items. Apparently, this method seems to be reliable to elucidate the real role of hosts in the life cycle rather than the simply passing of the developmental stages through the digestive tract, as recently stated during the detection of *S*. *calchasi* in *Accipiter cooperi* and *Buteo jamaicensis* from California, USA [see (23)]. On the other hand, Gjerde et al. (3) and Juozaityte-Ngugu et al. (11) mentioned that the DNA of *S. truncata* oocysts in *H*. *albicilla* from Norway and those of *Sarcocystis* spp. in corvid birds from Lithuania, respectively, were from stages, merely passing through the intestine of eagles rather than those produced in its intestinal mucosa. Gjerde et al. (3) suggested that the DNA of *S*. *truncata* identified through PCR and sequencing could either originate from oocysts/sporocysts in the intestine of a definitive host (cat, lynx) ingested by the white-tailed eagle or from sarcocysts in the muscle in an infected red deer carcass scavenged by the eagle. They considered the first option the most likely since they found a few oocysts that were much smaller than the majority. Moreover, lamina propria appears to be the site of infection most used for *Sarcocystis* spp. in *Accipiter* hawks (24, 25) and this study.

In the present case, scrapings of several spots of the intestinal mucosa and their subsequent molecular characterization allowed confirming the presence of single infection by *S*. *lutrae*. On the contrary, Gjerde et al. (3) found the white-tailed eagle to harbor three species of *Sarcocystis* in Norway, but only two of those (*S*. *halieti, S*. *lari*) used the sea eagle as a definitive host, thus showing that this host can play that role for more than one species. As already stated, the specificity of *Sarcocystis* spp. in birds of prey seems to be low and co-infections of congeneric taxa frequently occurred [see (3, 4, 23)].

Even though only one eagle was positively infected by *S*. *lutrae*, its role as a definitive host is clear, although the source of infection is unknown. The eagle primary feeds on fish, birds, and mammals (5–8), and, apparently, the latter were responsible for transferring the parasite, since some mustelid, canid, or procyonid mammals have been reported as part of its diet and as intermediate hosts of *S*. *lutrae* in several European countries (9, 10, 18–22). However, the eagle and other members of Accipitridae seem just to be one of the various potential definitive hosts of *S*. *lutrae*, like *S*. *halieti*, which uses *Accipiter nisus, H*. *albicilla*, and *Milvus milvus* as definitive hosts (3, 4). More intermediate hosts and different age classes of the eagle should be examined to elucidate the real prevalence, transmission routes, and ability of the parasite to solely infect it, as well as the importance of eagles in spreading the parasite. In fact, the eagle population has grown since 1978–1985 when, besides the already existing wild specimens, nine individuals were released in South Bohemia and subsequently colonized almost the entire country [see (26, 27)], and this trend might help in increasing the spreading of infection stages in a larger geographical area.

## Conclusion

This study confirmed that the white-tailed eagle might act as a definitive host of *S*. *lutrae*. Previous studies have established that this bird of prey (raptor) is also a definitive host of *S*. *arctica, S*. *halieti*, and *S*. *lari*, which use different carnivores and groups of birds as intermediate hosts.

## Data availability statement

The datasets presented in this study can be found in online repositories. The names of the repository/repositories and accession number(s) can be found below: https://www.ncbi.nlm.nih.gov/genbank/, ON796570; https://www.ncbi.nlm.nih.gov/genbank/, ON796572; https://www.ncbi.nlm.nih.gov/genbank/, ON806939; https://www.ncbi.nlm.nih.gov/genbank/, ON805825.

## Author contributions

OM conceived and designed the study, performed laboratory analyses, and analyzed data. OM and DG-S wrote the main manuscript. Both authors read and approved the final manuscript.

## Funding

Open access funding was provided by the Faculty of Agrobiology, Food and Natural Resources, Czech University of Life Sciences Prague.

## Conflict of interest

The authors declare that the research was conducted in the absence of any commercial or financial relationships that could be construed as a potential conflict of interest.

## Publisher's note

All claims expressed in this article are solely those of the authors and do not necessarily represent those of their affiliated organizations, or those of the publisher, the editors and the reviewers. Any product that may be evaluated in this article, or claim that may be made by its manufacturer, is not guaranteed or endorsed by the publisher.

## Abbreviations

bp: base pairs
cox1: Cytochrome c oxidase subunit 1
DNA: Deoxyribonucleic Acid
ITS1: Internal Transcribed Spacer 1
rRNA: Ribosomal Ribonucleic Acid.
